# The North West Adelaide Health Study: detailed methods and baseline segmentation of a cohort for selected chronic diseases

**DOI:** 10.1186/1742-5573-3-4

**Published:** 2006-04-12

**Authors:** Janet F Grant, Catherine R Chittleborough, Anne W Taylor, Eleonora Dal Grande, David H Wilson, Patrick J Phillips, Robert J Adams, Julianne Cheek, Kay Price, Tiffany Gill, Richard E Ruffin, the North West Adelaide Health Study Team

**Affiliations:** 1 Population Research & Outcome Studies Unit, South Australian Department of Health, 11 Hindmarsh Square, Adelaide, 5000, South Australia; 2 Health Observatory, Department of Medicine, The University of Adelaide, The Queen Elizabeth Hospital & Health Service, Woodville Road, Woodville, 5011, South Australia; 3 Endocrine and Diabetes Service, The Queen Elizabeth Hospital & Health Service, Woodville Road, Woodville, 5011, South Australia; 4 University of South Australia, City East Campus, North Terrace, Adelaide, 5000, South Australia

## Abstract

The North West Adelaide Health Study is a population-based biomedical cohort study investigating the prevalence of a number of chronic conditions and health-related risk factors along a continuum. This methodology may assist with evidence-based decisions for health policy makers and planners, and inform health professionals who are involved in chronic disease prevention and management, by providing a better description of people at risk of developing or already diagnosed with selected chronic conditions for more accurate targeting groups for health gain and improved health outcomes. Longitudinal data will provide information on progression of chronic conditions and allow description of those who move forward and back along the continuum over time.

Detailed methods are provided regarding the random recruitment and examination of a representative sample of participants (n = 4060), including the rationale for various processes and valuable lessons learnt. Self-reported and biomedical data were obtained on risk factors (smoking, alcohol consumption, physical activity, family history, body mass index, blood pressure, cholesterol) and chronic conditions (asthma, chronic obstructive pulmonary disease, diabetes) to classify participants according to their status along a continuum. Segmenting this population sample along a continuum showed that 71.5% had at least one risk factor for developing asthma, chronic obstructive pulmonary disease or diabetes. Almost one-fifth (18.8%) had been previously diagnosed with at least one of these chronic conditions, and an additional 3.9% had at least one of these conditions but had not been diagnosed.

This paper provides a novel opportunity to examine how a cohort study was born. It presents detailed methodology behind the selection, recruitment and examination of a cohort and how participants with selected chronic conditions can be segmented along a continuum that may assist with health promotion and health services planning.

## Introduction

In westernised countries, chronic diseases now account for approximately 60% of global deaths and almost half of the global burden of disease [1]. In Australia, chronic conditions such as diabetes, asthma, and chronic obstructive pulmonary disease (COPD) contribute significantly to the burden of disease, and are listed among the top ten leading causes of death [2]. Diabetes and asthma are recognized as Australian health priorities because of the significant impact they have on those people with the condition, their families, health professionals and the health system [3]. Large epidemiological cohort studies of randomly selected participants can assist in the fight against the global chronic disease epidemic by providing intelligence on the development of multiple outcomes and changing chronic disease risks over time, as well as the modification of lifestyle and risk factors [4]. The North West Adelaide Health Study (NWAHS) is a longitudinal cohort of over 4000 randomly selected adults aged 18 years and over, who live in the northern and western regions of Adelaide (total Adelaide population approximately 1 million), the capital of South Australia (total South Australian population approximately 1.3 million) [5,6].

A major challenge for epidemiologists and public health professionals is to paint the chronic disease picture in new and innovative ways that may galvanise action by researchers, governments and communities. Identifying and describing specific population groups at risk (the term "at risk" is used in this paper in the policy and colloquial sense, meaning "high risk") may assist in maximizing the effectiveness of strategies for the prevention, early detection, and management of chronic conditions. The chronic conditions focused on in Stage 1 of the NWAHS were asthma, COPD and diabetes. Whilst recognising the significant impact of cancer and infectious diseases on society, it was considered that these conditions are being well-researched and managed by specialised teams of health professionals within the State.

The NWAHS is a collaboration between the South Australian Department of Health, the Central Northern Adelaide Health Service (including The Queen Elizabeth Hospital & Health Service (TQEH) and the Lyell McEwin Health Service (LMHS) campuses), The University of Adelaide, the University of South Australia and the Institute of Medical & Veterinary Science (IMVS). The research team represents a range of disciplines including academic and clinical medicine, public health, epidemiology, social science and nursing, using quantitative and qualitative methodologies. Collaborative longitudinal studies such as this one enhance the nature of epidemiological research [7] because of the specialist knowledge contributed and the co-operation between researchers, health professionals, policy makers and the community, which contributes to scientific endeavour and productivity. The importance of these collaborations have been recognised and expanded, and the next stage of the study (in progress during 2004 and 2005) has led to alliances being sought with a leading demographer to map the distribution of chronic diseases, risk factors and biographic information, as well as medical practitioners in the fields of arthritis, obesity, mental health and cardiovascular disease – adding to established relationships with experts in asthma, COPD and diabetes. In recognition that health and disease are also influenced at both the individual and community level, and with a growing demand for information from a health inequalities perspective, social sciences such as sociology and anthropology are increasingly being used to view health in general, and prevention of disease in particular. The multidisciplinary nature of the study has incorporated this approach through qualitative investigations of participants' experience of being involved in the study, experiences of people living with chronic conditions [8], and information needs of people with diabetes [9].

The focus of this paper is two-pronged: a detailed description of Stage 1 of the study methodology (including design, sample techniques and clinic protocols), as well as a segmentation of the study's representative population sample according to stage of selected chronic diseases along a continuum. The more effectively those with specific conditions are described, the more precisely they can be targeted by designing policy and interventions that consider a range of appropriate characteristics to reduce the prevalence and risk of chronic conditions in the population.

### Analysis

#### Background and purpose of the study

The NWAHS was formulated in 1997 by Professor Richard Ruffin and Associate Professor David Wilson, in response to the lack of longitudinal biomedical data on chronic conditions in South Australia. Trend analyses of data from South Australian Health Omnibus Surveys (HOS) [10] in the 1990's were revealing increases in the prevalence of diabetes [11] and asthma [12]. These face-to-face interview surveys, whilst providing valuable representative population data from large clustered samples on the self-reported prevalence of diagnosed chronic conditions such as asthma and diabetes, did not provide cohort data on a single randomly selected population nor biomedically measured data that could be used to segment the population along the chronic disease continuum.

**Figure 1:**
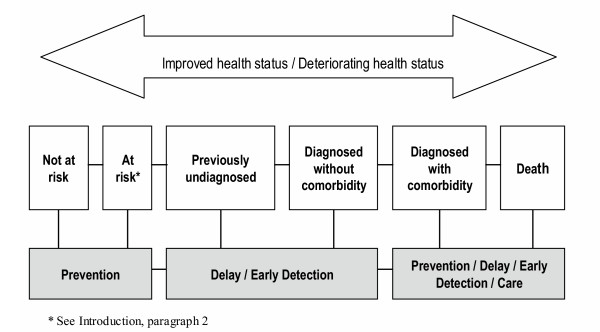
The chronic disease continuum.

The main aim of Stage 1 of the study cohort was to establish urgently needed baseline self-reported and biomedically measured information on diabetes, asthma and COPD and health-related risk factors in terms of a continuum, consisting of three main categories: those who were (1) at risk (see Introduction, paragraph 2) of these diseases; (2) previously undiagnosed with these diseases; and (3) previously diagnosed with those diseases (Figure 1). In addition to gaining insight into the management of chronic conditions and co-morbidities among those who have been diagnosed, identifying the additional categories of "at risk" (see Introduction, paragraph 2) and "previously undiagnosed" along the continuum provides a clearer statement of disease burden and epidemiological description of target groups, and presents opportunities for effective intervention, health service usage and health policy. Secondary aims from the longitudinal nature of the study include: (1) determination of incidence rates for a number of chronic diseases; (2) examination of changes in patterns of severity, management costs and utilisation of resources; (3) investigation of the impact of existing and future guidelines, including possible barriers to their implementation; and (4) provision of a high quality database for other health researchers to access for value-added chronic disease and/or risk factor related research. The NWAHS baseline data can also be used to compare reported and measured variables, particularly height and weight, as well as corroborate survey data regarding the prevalence of a number of health conditions and unhealthy behaviours among adults living the north western regions of Adelaide.

### Timeline

There were two phases involved in Stage 1 of the study, which involved the recruitment and baseline examination of the 4060 study participants: Phase 1A from December 1999 to December 2000 (n = 2523), and Phase 1B from August 2002 to July 2003 (n = 1537). Both phases used the same methodology for all aspects of the study, with the exception of an additional small number of questions in the telephone interview, the self-reported questionnaire and during the clinic examination (see Table 1).

**Table 1:** Comprehensive list of variables generated from data collection

Category	Variable	Measurement instrument
Demographics	Sex, age, contact details, occupation (kind of work done for most of life), number of adults (aged 18 years and over) and children in the household, reason for refusal (if applicable)	Recruitment (CATI) interview
	Age, country of birth, year of arrival in Australia (if applicable), gross annual household income, age when left school, highest educational qualification attained, marital status, work status, Aboriginal and/or Torres Strait Islander status, pension/benefit status	Self-administered questionnaire
Health conditions	Doctor-confirmed chronic disease status – diabetes, asthma, bronchitis, emphysema, heart attack, stroke, angina; anxiety, depression, a stress-related problem or any other mental health problem	Recruitment (CATI) interview
	Diabetes – doctor-confirmed prevalence, gestational diabetes, type of diabetes, time when first diagnosed (*Phase 1B only*)	Self-administered questionnaire
	Asthma, bronchitis, emphysema- doctor-confirmed prevalence; perceived severity of asthma, time when first diagnosed (*Phase 1B only*)	
Unhealthy behaviours	Smoking (type not specified)	Recruitment (CATI) interview
	Doctor-confirmed high cholesterol and high blood pressure, height and weight (*Phase 1B only*)	
	General health and well-being (SF-36), smoking (cigarette, cigar and/or pipe – current habit, number of cigarettes usually – now or ever – smoked in a day, age when last smoked (if relevant), and age when smoking first started (*Phase 1B only*), alcohol usage (frequency and number of standard drinks), physical activity (number of times within last two weeks, average time per exercise session and intensity – walking, moderate or vigorous)	Self-administered questionnaire
Family history and medical history	Family history of diabetes, heart disease and stroke; medical history of chronic health conditions – diabetes, asthma (including lung function), bronchitis, emphysema	Self-administered questionnaire
Health service utilisation	Number of all health (medical and surgical) services used during last 12 months (including general practitioner, hospital, allied health and alternative health)	Self-administered questionnaire
	Consent to link to Medicare and pharmaceutical information	Clinic examination
Biomedical measurements	Blood pressure, height, weight, waist and hip circumference	Clinic examination
Blood tests (fasting)	Triglycerides, total cholesterol, HDL and LDL cholesterol, glucose and HbA1c	Clinic examination
Skin allergy measurements	Saline (negative control), cockroach, house dust mite, cat dander, alternaria (mould), feather, rye grass, histamine [5%] (positive control)	Clinic examination
Lung function measurements	Pre and post FEV_1_, FVC, PEF (peak expiratory flow) and predicted percentages	Clinic examination

#### Selection of the sample population

Prior to the study commencing, approval for the research was obtained from the North West Adelaide Health Service Ethics of Human Research Committee. All households in the northern and western areas of Adelaide with a telephone connected and a telephone number listed in the Electronic White Pages (EWP) were eligible for selection in the study. The sample was stratified into two health regions: northern Adelaide and western Adelaide. The northern and western regions of Adelaide number approximately half of the city's population and one quarter of South Australia's population. These regions reflect the demographic profile of the State's population.

#### Recruitment

Participants were recruited using the telephone to conduct the interviews and the Electronic White Pages (EWP) as the sampling frame, which could be seen as a limitation of the study in that it may produce biased estimates because it excludes people who do not have a telephone connected or are not listed in the White Pages. However the alternative, random digit dialling (RDD), requires a significant amount of initial administrative work, can have lower response rates than EWP (often accessing business or other irrelevant telephone numbers) and is considerably more costly due to the number of calls required [13]. It has been shown, however, that there are no differences between random digit dialling and EWP in terms of the prevalence of chronic conditions [14]. In addition, people without their telephone number listed in the White Pages have been shown to be similar to those with a listed number in terms of reported health and risk factors [13]. Telephone surveys using CATI (Computer Assisted Telephone Interview) technology also have administrative and data collection advantages over other methods including automatic checks to reduce data entry errors, and the ability to conduct complicated questionnaire sequences based on previous responses [15].

**Figure 2:**
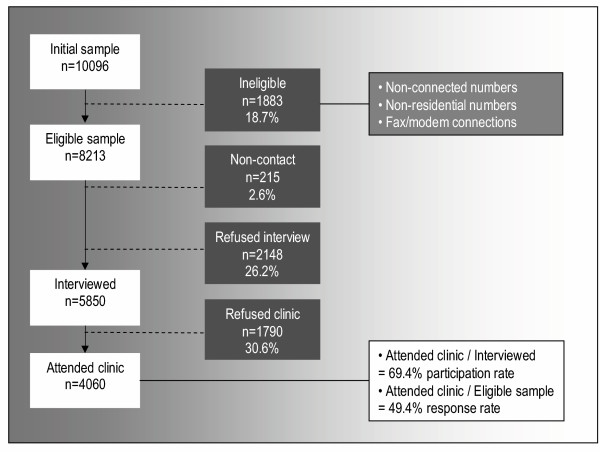
Response rates.

Within each household, the person who had their birthday last and was aged 18 years and over, was selected for interview and invited to attend the clinic for a biomedical examination. This method of randomly selecting within the household avoids bias towards unemployed and retired people or homemakers (often women) as those most likely to be home at the time that the interviews are conducted [16]. The response rate to the study is shown in Figure 2. To minimize potential bias due to differing probabilities of selection in the sample, the data were weighted by region (western and northern health regions), age group, sex and probability of selection in the household to the Australian Bureau of Statistics 1999 Estimated Resident Population [17] and the 2001 Census data [18].

Exclusion criteria were applied by recruiting staff at the initial telephone contact. Interviewers were responsible for determining if the selected respondent had sufficient intellectual ability to understand the implications and requirements of participating and if not, to thank the respondent for their time and terminate the interview. Those people who indicated that they were too ill to participate were similarly questioned by the interviewer to ascertain if they had sufficient physical abilities to attend the clinic, either using their own transport means or a taxi provided by the study. Both categories were coded by interviewers as "too sick" to participate in the study (n = 77). Of those who were eligible to the study and could be contacted, 2148 respondents (26.9%) refused any participation in the study (see Figure 2) due to a number of factors including being too busy, not wanting to participate, or considering themselves to be too old.

This cohort study did not recruit people residing in institutions, such as nursing homes, the majority of whom are elderly women, because of the inability to randomly select one individual from the group living arrangement. However people who had their own telephone number and who were living in individual units attached to a nursing home were eligible to participate. The study did not include those people from a non-English speaking background who could not communicate sufficiently well with the telephone interviewer and who could not answer questions at the initial recruitment stage, although every effort was made to encourage family members to assist in translating. A short trial of having an interpreter present if required for the clinic examination, was found to be neither time nor cost-effective. A review of non-participants found that only four people had refused to participate in the study due to the perceived language barrier. In addition, the small number of Aboriginal and/or Torres Strait Islander people (n = 20) recruited in this cohort means that no association or causality inferences can be made on their data because of the potential for misrepresentation, as highlighted by the Aboriginal Health Research Ethics Committee (South Australia).

To maximise the response rate, the telephone interview was restricted to approximately 15 minutes and a letter and information brochure, including endorsements by prominent South Australian sports people, were sent to the household of each selected telephone number, informing the household of the purpose of the study and indicating that they would be contacted by telephone about participating. Within two weeks of the letter and brochure mail-out, telephone calls were made to householders at various times of the day and evening, both during the week and on the weekend. The interviews were conducted in English by professional interviewers. Up to ten calls were made to each household in an effort to speak to someone. Repeated unsuccessful attempts that resulted in either no answer, the busy signal or a message being left on an answering machine were considered to be "non-contacts" and regarded in the same light as refusals (see Figure 2). Contact details supplied in the approach letter and brochure did however enable those participants who had not been contacted and who did want to take part to call the study co-ordinator to arrange a mutually convenient time for the telephone recruitment interview.

The study was pilot tested in November 1999 (n = 100) and recruitment was conducted between December 1999 and July 2003. During planning, the importance of ensuring a good response rate for the clinic visit resulted in various recruitment strategies being piloted to determine if remuneration or blood collection method influenced the response rate. Research from 1998 [19] suggested that a prepaid token monetary incentive, rather than a promised monetary or non-monetary 'reward', could have a positive effect on the response rate – although one study cited in this paper found that for "households not living in poverty, the $20 incentive payment increased the odds by a factor of just 1.1." (p24). This perceived benefit must be balanced against the increased costs of providing an incentive, particularly if it is applied to all eligible participants and not only those who ultimately take part. A subsequent pilot study of 58 people from the northern and western regions of Adelaide found that there was no statistical difference in response rates between those groups offered a financial incentive ($20) to attend the clinic (n = 29), and those groups who were not (n = 29); indeed, more people agreed to participate from the latter group (19 compared to 17). In addition, there was no difference found in response rates between those participants who had their blood sample taken at the clinic and those who attended the IMVS (a medical pathology service with a number of metropolitan and regional laboratories). It was therefore decided that no remuneration would be offered to study participants and that blood samples would be taken at both TQEH and LMHS sites.

Three qualitative sub-studies using focus groups were conducted during the first twelve months of the study on community responses to the notion of participating, as well as the notion of having participated and being unwilling to participate. While those who were unwilling to participate (n = 6) said they believed the study to be worthwhile, five said their primary reason for refusal was due to time constraints, and one did not believe in conventional medicine. Other contributing factors included a personal trauma about hospitals, doctors and needles, and a reluctance to deal with people generally. Neither the offer of transport to and from the clinic appointment nor a monetary incentive influenced their decision to participate.

A unique identification number (ID) was allocated to each household in the sample prior to the commencement of recruitment, and became the identifier for the participant. Initially this began with the number 1 through to 6,194, and did not differentiate between the western and northern regions, however the second and subsequent samples allocated 7,000 to 9,999 to the western regions and 10,000 to 11,999 to the northern regions which assisted the study team to immediately recognise which health service would be used. This ID number was used as much as possible to replace identifying details such as name and address on much of the participant's information, to protect confidentiality and ensure anonymity with stored data.

The Questionnaire Programming Language (QPL) system was used to conduct the recruitment interviews [20]. This is a "freeware" package that provides an efficient and reliable process of automating survey data by allowing immediate entry of data from the interviewer's questionnaire screen to the computer database. Its main advantage is that it correctly sequences questions as specific answers are given, allowing skips to another section or prompts to be incorporated. In addition, it enforces a range of checks on each response with most questions having a set of pre-determined response categories. The QPL program also allows open-ended responses to be transcribed verbatim by the interviewer.

The same three recruiting staff were used for the telephone interviews, to establish and maintain a rapport with eligible participants, particularly if return telephone calls to rebook cancelled appointments were needed. These staff developed an in-depth knowledge of the study, having participated in the clinic examination prior to the study starting, and were able to build upon this knowledge through regular meetings with the study management team.

During the recruitment interview, respondents were asked a number of health-related questions including doctor-confirmed chronic medical (diabetes, asthma, bronchitis, emphysema, heart attack, stroke and angina) and mental health disease status, and smoking. Additionally in Phase 1B, participants were asked about being told by a doctor or nurse that they have high cholesterol and/or high blood pressure, and if they still have either of these conditions (Table 1 and Appendix B). They were further asked for their height and weight to enable comparison with the actual measurement recorded at the clinic, for a nested sub-study that examined the accuracy of self-reported versus measured height and weight. Reasons for non-participation were also asked of those who refused. Information was collected on the number of adults and children (aged less than 18 years of age) living in the household, to determine the correct figures for later weighting of the sample to correct selection bias. The respondent's age was requested, rather than their date of birth, due to the sensitive nature of this information and the reluctance of many people to divulge this information over the telephone. Information regarding the respondent's sex was collected for verification with their name (particularly with androgynous given names), and the kind of work they had undertaken for most of their life was collected for the purposes of social epidemiological analyses.

Respondents were invited to make an appointment for a biomedical examination at TQEH (western region) or the LMHS (northern region). Participants were sent an information folder containing a letter confirming the appointment date and time, a questionnaire to complete and bring to the clinic, an information sheet, a map of the clinic, a study fridge magnet, a form for advising change of details, information about child care facilities, and reminders including the need to fast for eight hours before their appointment. Study management staff also prepared a wallet for each participant for use in the clinic, with pre-prepared consent forms and a clinic running sheet. This wallet was labelled with the participant's ID number, labelled with a coloured sticker if the participant attended the LMHS (to enable easy sorting and filing) and is used for storage of relevant study information.

#### Self-reported questionnaire

The self-reported questionnaire contained questions on demographic and health-related issues (Table 1 and Appendix B). Many of the questions and categories used in this questionnaire were taken from the South Australian HOS questionnaires, to enable comparisons to be made with prevalence of selected chronic conditions and health related risk factors in the overall South Australian population. Previously diagnosed diabetes status and type, asthma status and severity, and status of COPD were determined. The questionnaire also included the Chronic Lung Disease Index [21].

Questions also included a number of health-related risk factor behaviours (see questionnaire, Appendix B). To ascertain their personal alcohol risk, respondents were asked the number of standard drinks they usually have on a weekly and daily basis. Participants were asked if they had ever regularly or currently smoked: if so, how many cigarettes had been or were usually smoked a day, or if they only smoked cigars or pipes; and at the ages at which they first started smoking and if appropriate, when they last gave up smoking. Physical activity was based on questions regarding the number of times in the last two weeks and total amount of time spent walking for exercise, and performing moderate and vigorous exercise.

Quality of life was assessed using the Short Form 36 (SF-36), a generic instrument validated for use in Australia [22]. The General Health Questionnaire 28 (GHQ-28) was asked of a sub-sample (n = 1240) of participants [23] but dropped after feedback was received that it was too onerous for respondents to complete in addition to the SF-36. Utilisation of health care services such as general practitioner, community health centre, day surgery, specialist and allied health professionals was ascertained, and demographic questions were included as listed in Table 1.

### Biomedical examination

#### Consent and administrative information

Clinic appointments of approximately one-hour duration were held from Monday to Saturday (to allow for people who work full time to attend). At each appointment, the participant was given additional detailed information about the study and asked to sign consent forms for participation in the study. The information given highlighted the longitudinal nature of the study, and participants were informed that they may be invited to participate in health-related sub-studies. These included qualitative work that focused on participants' experiences of living with and managing chronic conditions [8], as well as exploring goals and projects to maintain the sustainability of the health and well-being of the communities involved.

Participants were also asked for secondary contact details that would allow study staff to follow-up their whereabouts if they changed address and/or telephone details, and for details of their general practitioners for notification of their results. A result letter detailing a number of the clinic measurements was provided to the participant as feedback on their health, serving as a tangible benefit for participating.

Most Australians have been issued with either an individual or family Medicare number, which is used at each medical or hospital occasion of service. Participants were asked to provide consent for release of their retrospective and prospective medical and hospital-related information via Medicare over an eight year period, for health economic-related and pharmaceutical use analyses. Medicare is Australia's universal system for financing services provided by private doctors, public hospitals and some additional health costs, and is administered by the Federal Government. Medicare collects national data on medical provider service claims for payment purposes through the Medicare Benefit Schedule, and therefore captures the majority of significant occasions of health service use. It has been shown to be more accurate than self-reported service utilization data [24,25]. Medicare also collects national data on medications prescribed through the Pharmaceutical Benefits Scheme. Results of analyses of health service and pharmaceutical use of study participants will be reported independently.

### Biomedical measurements

Biomedical measurements began with blood pressure readings, using a standard, calibrated blood pressure sphygmomanometer. Two blood pressure measurements were taken five to ten minutes apart while the participant was relaxed and seated. The average of these two recorded measures were used. Height without shoes was measured to the nearest 0.5 centimetres using a wall-mounted stadiometer (height measurement), and weight to the nearest 0.1 kilogram in light clothing and without shoes using standard digital scales. Waist circumference was measured to the nearest 0.1 centimetre using an inelastic tape maintained in a horizontal plane, with the subject standing comfortably with weight distributed evenly on both feet. The measurement was taken at the level of the narrowest part of the waist. Hip circumference was also measured using an inelastic tape, at the level of the maximum posterior extension of the buttocks. Three measurements of the waist and hip were taken and the mean for each was calculated.

A fasting blood sample of approximately 10 ml was taken to measure triglyceride, total cholesterol, high density lipoprotein (HDL), low density lipoprotein (LDL), glucose and glycosylated haemoglobin (HbA1c) levels.

Skin allergy tests were conducted in relation to asthma to determine reactions to the following allergens: saline (negative control), histamine 5% (positive control), rye grass, cat dander, house dust mite, alternaria (mould), feather and cockroach. The test involved pricking the forearm skin with a lancet via a drop of allergen. The diameter of the skin wheal was measured (in millimetres) both vertically and horizontally at 15 minutes, and the two measurements were averaged.

Lung function tests were performed using a Microlab 2000 spirometer both pre- and post-salbutomol administration [26]. The tests involved participants being asked to sit upright, put their feet firmly on the floor, and have a nose clip applied. They were then asked to take a deep breath through their mouth to total lung capacity, to seal their lips around the mouthpiece of the spirometer and then to forcefully expel the breath through the mouthpiece as fast and as hard as they could to residual volume. Once two acceptable spirograms had been registered, participants were administered 400 μg of salbutomol via a metered dose inhaler (MDI) and a spacer. Another two acceptable spirograms were performed ten minutes after the salbutomol.

A summary of the data collected from participants in the recruitment interview, self-completed questionnaire, and the biomedical examination is listed in Table 1, with the data collection forms in the appendices. The study team cast a wide net to gather baseline information across a range of health outcomes, risk factors and determinants.

For quality control purposes, following the completion of Phase 1A, a telephone interview of a random selection of 340 participants for an exit study was conducted to explore the experiences, perceptions and satisfaction regarding the recruitment process, information folder and clinic examination. All provided valuable feedback for conducting the remainder of the study, and suggestions (such as needing more space in the clinic) lead to improvements in clinic accommodation.

### Establishing and maintaining a relationship with the study cohort

Several strategies to encourage participation and maintain contact with participants over the long-term were employed, based on previous successful longitudinal studies such as the Framingham Heart Study, the Busselton (Western Australia) Study and the Women's Health (Australia) Study.

The use of an easily recognizable study logo (designed in-house and incorporating the State's floral emblem, Sturt's Desert Pea) on all items associated with the study including brochures, forms, correspondence, reports, conference presentations and posters, and a fridge magnet promoted the study's identity among the study team, participants, and the wider health and research community.

In the initial approach, potential participants were invited to assist both themselves and their community to improve their health, as well as contribute to important health knowledge that would help their region lead the way in community health care in Australia. A second round of brochures included with the approach letter sent to participants recruited towards the middle and end of the study, included favourable comments made by existing participants during an exit study. This theme was repeated in the December 2003 newsletter with the use of quotations from participants in the Framingham Heart Study, commenting how they not only valued the regular free health check, but also the altruistic benefits of the study. These sentiments and the importance of their ongoing participation (especially in light of the inability to substitute other people if they withdrew) are echoed by recruitment, study research and front-line clinic staff in all verbal and written communications with participants. Participants were further reassured in study materials of the confidentiality of their personal and medical information given, and its de-identification prior to being used for research purposes, as well as their ability to withdraw from the study without change to their health care from the services involved.

In addition to annual newsletters and notification of their individual clinic results, participants are also sent birthday cards as a means of maintaining contact and providing them with an opportunity to inform the study team of any changes in their contact details. Participants have reported favourable comments and expressions of appreciation about these communications to study team and clinic staff during routine clinic visits and follow-up telephone calls. As another means of maintaining contact with the cohort, a telephone questionnaire was conducted with Phase 1A participants (response rate 91.7%) in April 2002, which also served to provide information about whether participants followed up clinic results with general practitioners, and if there were any changes in personal circumstances, as well as a number of questions relating to asthma and diabetes. Two local functions were also held for study participants, the study team, and local and State government dignitaries, to launch the key biomedical findings from Phase 1A.

While mail and telephone remain the major forms of communication with participants, the study team has recognised the growing trend towards use of home computers and the World Wide Web, with approximately one quarter of the Australian population (over 5 million people) reporting use of the internet including over 30% of people aged 55 years and over [27]. Therefore, to assist with dissemination to study participants, and also the wider Australian community and health professionals worldwide, a dedicated website  was developed, highlighting aspects of the study including the study team, results, and frequently asked questions. The website also provides links to all study reports, and conference presentations and posters, as well as an online enquiry form that generates an email to the study team, for questions or notification of change of contact details.

Tracking of participants has included the use of the White Pages telephone directory, the following up of secondary contacts and the State Electoral Roll. The database of all deaths registered in South Australia through the Births, Deaths and Marriages Registration Office has been used to search for possible matches with the study cohort, and plans are being made to access the National Death Index for possible deaths of study participants now living interstate.

### Lessons learnt so far

Lessons learnt from Stage 1 of the study and technological developments since then, resulted in the following improvements being implemented for Stage 2 of the study (undertaken from May 2004 to February 2006):

• The use of hand held computers (hp iPAQ Pocket PC h4000 series) rather than paper-based forms for data entry of clinic measurements: this allows for immediate cleaning and analysis at the completion of the study, as well as baseline measurements to be fed back to participants at the clinic visit. These computers are also used by clinic staff to enter subsequent biomedical results from the blood and lung function tests as back-up tasks when participants do not attend their appointment. The initial financial outlay more than justified the ease of use by clinic staff, the immediacy of data transfer for research staff as well as result letters to participants and their doctors, and the improved storage and confidentiality of data.

• Use of home and nursing home visits for participants whose health has deteriorated since Stage 1.

• The collection of information about all medications being taken (including vitamins, patches and contraceptives): this will provide information about contraindications and possible effects on diagnosed and undiagnosed conditions and risk factors.

• The collection of both self-reported and biomedical measurement of some subjects (eg height and weight): this will be used to verify self-reporting behaviour such as the tendency to over-report height and under-report weight, as well as identify population groups who may believe their condition is being sufficiently well-managed, but whose biomedical results belie this.

• The storage of a blood sample from each participant for further testing: tests are yet to be determined but may include the measurement of Vitamin D and parathyroid hormones which are linked to osteoporosis, and c-reactive protein in relation to arthritis and coronary heart disease.

### Future plans for the cohort

Future biomedical measurement of the cohort is planned every three years, with an interim follow-up telephone questionnaire scheduled for 2007 to maintain contact with participants and for data collection purposes. Stage 3 (which is scheduled to occur in 2008–09) would benefit from the further use of technology to scan self-report questionnaires and the possible use of automatic SMS texting to mobile telephones for clinic visit reminders. Over one-third of our participants have provided their mobile telephone numbers, and email addresses are also being collected in recognition of these modern and frequently-used forms of communication.

Additional chronic conditions as yet to be investigated include sleep apnoea and incontinence. Nutrition is another health-related risk factor for which data could be collected in future stages. The collection of consent for linkage with state-based hospital databases, as well as additional information regarding the participant's past history for life-course analysis, are being considered as future data collection items. In hindsight, information regarding the age of death from heart disease (if <50 years of age) of first generation family members would have provided valuable information regarding the risk of developing heart diseases. Valuable lessons can be learnt from the Framingham Heart Study for their specialist targeted recruitment of ethnic populations to address the minorities within the community, and in their recruitment of participants' spouses and children for genetic/family links regarding chronic disease. Genetic testing of NWAHS participants may be considered in the future.

### Description of cohort

Table 4 provides the demographic profile of the study participants (n = 4060), including highest level of education achieved, annual gross household income, marital and work status.

### Definitions of chronic conditions and health-related risk factors

Definitions and cut-off points for the chronic conditions and related risk factors were drawn from a range of accepted local, national and international evidence-based guidelines and standards. Subsequent analyses for report writing, presentations and publications are adapted to suit the targeted audience. The following definitions and cut-off points were used in this paper for the purposes of classifying study participants along the chronic disease continuum.

Alcohol risk was then calculated based on the 1989 National Heart Foundation Risk Factor Prevalence study classification formulae [28]. This criterion was selected to enable comparisons with past HOS questionnaires. Respondents were categorised as non-drinkers, no risk drinkers, low risk drinkers, intermediate risk drinkers, high risk drinkers and very high risk drinkers (Table 2, Table 3). These categories were further collapsed into non drinkers or no risk drinkers; low risk; and intermediate to very high risk for analysis.

**Table 2:** Alcohol risk: categories of risk level [26]

Frequency of drinking	Number of drinks					
	1–2	3–4	5–8	9–12	13–20	>20
Less than once a week	B	B	B	C	D	E
1 or 2 days	B	B	B	C	D	E
3 or 4 days	B	B	C	D	E	F
5 or 6 days	B	C	D	E	F	F
Every day	B	C	D	E	F	F

**Table 3:** Alcohol risk levels [26]

Category	Description	Risk	
		Men	Women
A	Non-drinkers	None	None
B	Average daily intake of less than 3 drinks	None	Low
C	Average daily intake of 4 drinks or 9–12 drinks in any day	Low	Intermediate
D	Average daily intake of 5–8 drinks or occasional excess	Intermediate	High
E	Average daily intake of 9–12 drinks or frequent or great occasional excessive intake	High	Very high
F	Average daily intake of over 12 drinks	Very high	Very high

**Table 4:** Profile of participants

Variable	n	%
Sex		
Male	1988	49.0
Female	2072	51.0
Age group (years)		
18 – 34	1411	34.8
35 – 54	1437	35.4
55 – 74	878	21.6
75 +	335	8.2
Area of residence		
Western suburbs	1853	45.6
Northern suburbs	2207	54.4
Highest education level obtained		
Secondary	1751	43.1
Trade/Apprenticeship/Certificate/Diploma	1641	40.4
Bachelor degree or higher	475	11.7
Not stated	193	4.8
Gross household income		
Up to $20,000	902	22.2
$20,001–40,000	1008	24.8
$40,001–60,000	899	22.2
More than $60,000	992	24.4
Not stated	258	6.4
Aboriginal or Torres Strait Islander origin		
Yes	20	0.5
No	3548	87.4
Not stated	492	12.1
Country of birth		
Australia	2865	70.6
UK or Ireland	645	15.9
Europe, Asia & Other	524	12.9
Not stated	25	0.6
Marital status		
Married or living with partner	2525	62.2
Separated/Divorced	331	8.1
Widowed	232	5.7
Never married	940	23.1
Not stated	32	0.8
Work status		
Full time employed	1537	37.9
Part time/Casual employed	728	17.9
Unemployed	173	4.3
Home duties/Retired	1239	30.5
Student/Other	333	8.2
Not stated	49	1.2
Receive pension from Department of Social Security		
Yes	1286	31.7
No	2698	66.5
Don't know/Not stated	75	1.8

Total	4060	100.0

Participants were classified as non-smokers, ex-smokers or current smokers based on their responses to questions about current and past regular habits of smoking cigarettes, cigars or pipes. Their level of smoking (non-smoker, ex-smoker, light, moderate and heavy) was determined by the number of cigarettes smoked per day; if they indicated that they smoked only cigars or pipes, they were considered to be light smokers. These questions were also included to enable comparison with past HOS questionnaires, used to monitor the state's smoking prevalence by The Cancer Council South Australia.

The physical activity questions from the Australian National Health Surveys (conducted in 1989/90, 1995 and 2001) were used in this study to also enable comparison with national data – the now recommended Active Australia questions had not been formulated when the study was initiated. Physical activity level was based on a score derived from the formula "***e ***× ***t ***× ***i***"" where ***e ***was number of times walking, moderate and/or vigorous exercise was undertaken during the past two weeks, ***t ***was the average amount of time spent on each exercise session and ***i ***was the intensity (walking scored at 3.5, moderate exercise scored at 5.0 or vigorous exercise scored at 7.5). Participants were classified thus: sedentary (score less than 100, including no exercise), low (score of at least 100 but less than 1600), moderate (score of at least 1600 to 3200, or more than 3200 but less than 2 hours of vigorous exercise) and high (score of at least 3200 and 2 hours or more of vigorous exercise) [29].

High blood pressure was defined as systolic blood pressure greater than or equal to 140 mmHg and/or diastolic blood pressure greater or equal to 90 mmHg [28,30]. Body mass index (BMI) was calculated as weight (in kilograms) divided by height(in metres)^2^. Overweight was defined as BMI ≥ 25.0 and obesity as BMI ≥ 30.0 [31]. A waist circumference (WC) of ≥ 95 cm for males or ≥ 80 cm for females was defined as the level at which no further weight should be gained. A WC of ≥ 100 cm for males and ≥ 90 cm for females was defined as the level at which weight reduction should be recommended [32,33]. A high waist-hip ratio was defined as > 1.0 for males and > 0.85 for females [34].

High cholesterol was defined as total cholesterol greater than or equal to 5.5 mmol/L [35]. Impaired fasting glucose (IFG) was defined as fasting plasma glucose (FPG) of at least 6.1 mmol/L and less than 7.0 mmol/L [36]. Participants were considered to have undiagnosed diabetes if their FPG was greater than or equal to 7.0 mmol/L and they did not report being told by a doctor that they had diabetes.

A skin allergy test was considered to be successful if the participant's histamine (positive control) wheal was greater than 2 mm in diameter. Participants were defined as having an allergic reaction to the selected allergen if the skin wheal was greater than the negative control by 2 mm or more in diameter.

Participants were considered to have asthma if they experienced at least a 15% increase in their forced expiratory volume in one second (FEV_1_) from pre-Ventolin to post-Ventolin; if they had at least a 12% increase in FEV_1 _from pre-Ventolin to post-Ventolin and their absolute difference in FEV_1 _was greater than 200 ml; or if they reported being told by a doctor that they had asthma and that it was current.

Participants were considered to have COPD if their measured FEV1:FVC (forced vital capacity) ratio was less than the result of the formula (87.21 - (0.18 × age) × 0.882) for males, and (89.10 - (0.19 × age) × 0.893) for females [37,38]. In this formula, 0.882 and 0.893 represent one minus two standard deviations from the predicted mean for males and females, respectively. People with previously undiagnosed COPD were defined as having COPD according to the above European Respiratory Society criteria, but who did not report having been told by a doctor that they had COPD (chronic bronchitis or emphysema).

### Data analysis

Data were weighted to the 1999 Estimated Resident Population and 2001 Census [17,18], the Australian Bureau of Statistics official measures of the Australian resident population, by age group, sex, region and probability of selection in the household, to ensure that the sample was representative of the population in the northern and western regions of Adelaide. The data were analysed using SPSS (Version 10.0) [38].

### Segmentation of the cohort for selected chronic conditions

Table 5 provides an initial segmentation of the cohort's baseline chronic condition status along a continuum (Figure 1). Participants were defined as "not at risk" (Figure 1) if they did not have any of the chronic conditions: that is, they did not have asthma, COPD or diabetes, nor a self-reported doctor diagnosis of heart attack, stroke or angina, nor did they have any risk factors (obesity, high waist/hip ratio, current smoker, high blood pressure, high total cholesterol, a sedentary physical activity level, intermediate to very high alcohol risk, gestational diabetes, or skin allergies). Those in the category "at risk" (see Introduction, paragraph 2) were considered to have either IFG, and/or one or more of the above risk factors. Participants allocated to the category "previously undiagnosed" were considered to have no self-reported doctor diagnosis of asthma, COPD or diabetes but one or more of these conditions detected at the clinic. Participants "diagnosed with or without comorbidity" were considered to have a self-reported doctor diagnosis of asthma, COPD or diabetes and/or a self-reported doctor diagnosis of either heart attack, stroke or angina.

**Table 5:** Segmentation of study cohort for selected chronic diseases – asthma, COPD and diabetes

	**n**	**%**	**(95% CI)**
Not at risk of chronic disease	234	5.8	(5.1 – 6.6)
At risk of chronic disease	2871	71.5	(70.1 – 72.9)
Undiagnosed chronic disease	156	3.9	(3.3 – 4.5)
Diagnosed chronic disease	755	18.8	(17.6 – 20.0)

Total	4015*	100.0	

*45 cases excluded due to missing responses in questionnaire or insufficient FEV1 or FVC results

^# ^See Introduction, paragraph 2

NB: Chronic diseases – asthma, COPD, diabetes. Details of categories are to be found in "Classification of continuum". Where overlap occurred between categories, participant included in more serious category, ie person with undiagnosed asthma and self-reported diabetes considered to be in "Diagnosed chronic disease" category.

The distribution of participants in the chronic disease continuum shows that almost one in five people (18.8%) had a self-reported doctor diagnosis of either diabetes, asthma, COPD, heart attack, stroke and/or angina. Overall, 5.8% had no risk factors for chronic disease. Almost three out of four people (71.5%) did not have any of the chronic conditions of interest (undiagnosed or diagnosed) but had at least one risk factor that may lead to one or more of these conditions developing.

## Conclusion

The NWAHS has provided valuable baseline evidence through its use of a continuum for selected chronic diseases. South Australia has an ageing population and the highest elderly population of all the states and territories in Australia, with 13.8% aged 65 years and over [39]. These facts are important given the association of age with many chronic conditions, health service use and costs.

This cohort study provides a unique opportunity to provide much needed measured and self-reported information to fill identified gaps in the study of chronic diseases and risk factors. Its sound epidemiological base, large random sample and reasonable response rate allow its generalisability, firstly for the local community it represents, secondly for South Australians and thirdly, for the wider Australian population, whilst providing useful research results for the international public health community. The longitudinal dimension of the study has been realised with participants having returned to the clinic for their follow-up visit in 2004–06. This will ensure provision of rich and varied data that should contribute to the overall improvement of health and well-being among present and future generations, and will be the subject of a future paper.

In addition to the baseline prevalence data that the NWAHS has provided, the longitudinal nature of the study will, in time, enable the natural history of conditions to be examined. The characteristics of people who moved to the right along the disease continuum to worse states of health over time will be compared to those who do not move. Valuable lessons for health promotion may be learned from those participants who moved left along the continuum, to an improved state of health. Viewing health and disease as a continuum in this study means that recommendations for health interventions, planning and policy will be focused on prevention and early intervention of risk factors and disease, rather than just management and delay of chronic conditions.

Both Stage 1 and 2 of the study have been funded by academic and government research grants, reducing certain conflicts of interest that might result from sponsorship by large corporations, such as pharmaceutical companies.

The NWAHS is in a unique position to provide valuable baseline biomedical and self-reported data on a range of health conditions and their related risk factors, pertaining to both men and women aged 18 years and over, and promises to deliver follow-up evidence-based information that will grow in value as each examination is undertaken.

## Abbreviations

BMI Body Mass Index

CATI Computer Assisted Telephone Interview

COPD Chronic Obstructive Pulmonary Disease

EWP Electronic White Pages

FEV_1 _Forced Expiratory Volume in one second

FVC Forced Vital Capacity

GHQ-28 General Health Questionnaire

HbA1c Glycosylated haemoglobin

HDL High Density Lipoprotein

IFG Impaired Fasting Glucose

IMVS Institute of Medical and Veterinary Science (South Australia)

LDL Low Density Lipoprotein

LMHS Lyell McEwin Health Service (South Australia)

MDI Metered-Dose Inhaler

NWAHS North West Adelaide Health Study

QPL Questionnaire Programming Language

PEF Peak Expiratory Flow

SF-36 Short Form 36 (Questionnaire)

TQEH The Queen Elizabeth Hospital & Health Service (South Australia)

WC Waist Circumference

## Competing interests

The author(s) declare that they have no competing interests.

## Appendix

### Appendix A

The following persons comprised the study team for Stage 1 of the North West Adelaide Health Study 2000–2003:

Principal/Chief Investigators: R Ruffin, A Taylor, D Wilson, J Cheek, P Phillips, R Adams, K Price Co-Investigators/Collaborators: N Potts, P Zalewski, W Jeffries, C Beng

Research/Study Management: E Dal Grande, C Chittleborough, J Grant, T Gill, S Appleton, C Oster, K Baldock, B Hurst, L Caudle, M Burke, J Hickling, J Paynter

Clinic Co-ordination: I Meagher, S Pickering

Clinic: E Jansen, M Taylor, R Battersby, N Labiszewski, A Scardigno J Brown, A O'Grady, J Middleton, A Brindley, L Kovalenko, J Barnett

Recruitment:J Dibble, K Smith, B Webb, S Ogilvy, L Parry, D Hart.

### Appendix B

PDF documents: Telephone recruitment questionnaire, Self-report questionnaire, Clinic running sheet
